# Petrocarbon evolution: Ramped pyrolysis/oxidation and isotopic studies of contaminated oil sediments from the Deepwater Horizon oil spill in the Gulf of Mexico

**DOI:** 10.1371/journal.pone.0212433

**Published:** 2019-02-28

**Authors:** Kelsey L. Rogers, Samantha H. Bosman, Mary Lardie-Gaylord, Ann McNichol, Brad E. Rosenheim, Joseph P. Montoya, Jeffrey P. Chanton

**Affiliations:** 1 Department of Earth, Ocean and Atmospheric Science, Florida State University, Tallahassee, Florida, United States of America; 2 NOSAMS, Woods Hole Oceanographic Institute, Woods Hole, Massachusetts, United States of America; 3 College of Marine Science, University of South Florida, St. Petersburg, Florida, United States of America; 4 School of Biological Sciences, Georgia Institute of Technology, Atlanta, Florida, United States of America; University of Maryland Center for Environmental Science, UNITED STATES

## Abstract

Hydrocarbons released during the Deepwater Horizon (DWH) oil spill weathered due to exposure to oxygen, light, and microbes. During weathering, the hydrocarbons’ reactivity and lability was altered, but it remained identifiable as “petrocarbon” due to its retention of the distinctive isotope signatures (^14^C and ^13^C) of petroleum. Relative to the initial estimates of the quantity of oil-residue deposited in Gulf sediments based on 2010–2011 data, the overall coverage and quantity of the fossil carbon on the seafloor has been attenuated. To analyze recovery of oil contaminated deep-sea sediments in the northern Gulf of Mexico we tracked the carbon isotopic composition (^13^C and ^14^C, radiocarbon) of bulk sedimentary organic carbon through time at 4 sites. Using ramped pyrolysis/oxidation, we determined the thermochemical stability of sediment organic matter at 5 sites, two of these in time series. There were clear differences between crude oil (which decomposed at a lower temperature during ramped oxidation), natural hydrocarbon seep sediment (decomposing at a higher temperature; Δ^14^C = -912‰) and our control site (decomposing at a moderate temperature; Δ^14^C = -189‰), in both the stability (ability to withstand ramped temperatures in oxic conditions) and carbon isotope signatures. We observed recovery toward our control site bulk Δ^14^C composition at sites further from the wellhead in ~4 years, whereas sites in closer proximity had longer recovery times. The thermographs also indicated temporal changes in the composition of contaminated sediment, with shifts towards higher temperature CO_2_ evolution over time at a site near the wellhead, and loss of higher temperature CO_2_ peaks at a more distant site.

## Introduction

The results of a number of field studies indicate unambiguously that oil residues from the Deepwater Horizon (DWH) oil spill were deposited on the seafloor [1–7]. Of the total oil released, an estimated 0.5–14.4% was deposited on the seafloor [1,3]. Passow and Ziervogel [8] argued that these estimates were low because they failed to consider the formation of marine oil snow over the total spread of the surface oil slicks, which could have resulted in a greater extent of seafloor deposition. The bulk of the sedimented oil-residue was limited to the surface sediment as defined by radiocarbon [3], hopane [1,4], and other radioisotopes [2].

The severity of impacts on benthic communities depends on the nature of the petroleum-derived material which was deposited on the seafloor. It has been suggested that biodegradation and dissolution of oil in the water column prior to deposition on the seafloor moderated these impacts [5,7,9]. We used ramped pyrolysis oxidation (RPO) to assess the biodegradation state of the material present on the seafloor due to the blowout. With RPO we examined 5 sites in all, 3 contaminated sites, two in time series and one as a function of depth, a control uncontaminated site and a natural seep site. Several studies have analyzed the recovery of contaminated sediments and have shown a reduction in the overall extent of contamination and have estimated degradation rates. Stout et al. [4] and Adhikari et al. [10] showed reduced coverage of elevated levels of hopane and polycyclic aromatic hydrocarbons (PAHs) in the years following the blowout. Studies by Stout and Payne [5] and Bagby et al. [9] analyzed biodegradation rates of multiple hydrocarbons in the sediment, showing that biodegradation continued on the seafloor after the deposition of the sedimented oil-residues. In contrast to focusing on specific petroleum compounds, studies by Pendergraft et al. [11] and Pendergraft and Rosenheim [12] employed ramped pyrolysis/oxidation paired with carbon isotope analysis on bulk coastal sediments. We applied their approach to the deep-sea floor.

RPO is an approach to determine the thermochemical stability of organic matter [13]. When paired with δ^13^C and Δ^14^C isotopic analysis, the source of the carbon can be inferred as a function of thermal stability. The thermochemical stability of a compound is based on the amount of energy needed to break the bonds, with higher stability requiring higher temperatures, whereas more labile bonds break at lower temperatures. The thermal stability of a compound is thus related to its lability, reactivity, and suitability as a substrate in microbially mediated reactions [14]. Fresh crude oil is quite labile, oxidizing at relatively lower temperatures [12]. Oil degradation leads to oxygenated and higher molecular weight compounds that oxidize at higher temperatures [15]. Shortly after the DWH event, the oil released into the environment was oxygenated [16], consumed by a variety of microbes and likely converted to biomass, burned, or altered in many ways [15,17–19]. We define this altered and unaltered petroleum-based product as petrocarbon [3]. Since portions of this material are no longer amenable to gas chromatographic separation and analysis [15], the best method to identify it is isotopically, specifically with radiocarbon [20–22]

Pendergraft et al. [11] linked PAHs, an independent oil tracer, to changes in thermographs (temperature evolution of oxidized products as measured with an infrared gas analyzer) produced from oil contaminated marsh sediments. They found that sediments with elevated PAH content produced different CO_2_ thermographs with C isotope signatures indicating the presence of oil. Pendergraft and Rosenheim [12] studied the evolution of organic carbon over time in oil contaminated marsh sediments using RPO. The thermographs shifted from lower to higher thermochemical stability and corresponding isotopic signatures indicated increasing enrichment in both δ^13^C and Δ^14^C over time, indicating a transformation of the oil as it degraded in situ. These two studies show the ability of RPO to detect oil contamination through both the thermographs and isotopic analysis of the individual CO_2_ fractions.

The purpose of this study is to analyze the evolution of the carbon isotopic composition of bulk organic carbon over time and the change in thermochemical composition of sediments at 5 deep-water sites in the northern Gulf of Mexico. Oil deposition following the DWH oil spill was indicated by radiocarbon depletion in the bulk organic matter in the surface layer (0-1cm) of sediment [3]. With RPO we examined 5 sites: 3 contaminated sites, two in time series and one as a function of depth, a control site, and a natural seep site. We hypothesized that: 1) over time, the bulk isotopic composition of the surface layer in contaminated areas would return to baseline values, 2) the oil-residue deposited in the sediment following the DWH oil spill would be more thermally stable than fresh Macondo oil and 3) that over time and depth the oil residue would evolve towards greater thermochemical stability. The carbon isotope signatures of the RPO splits were used to infer the origin of the organic material.

## Materials and methods

**Fig 1:**
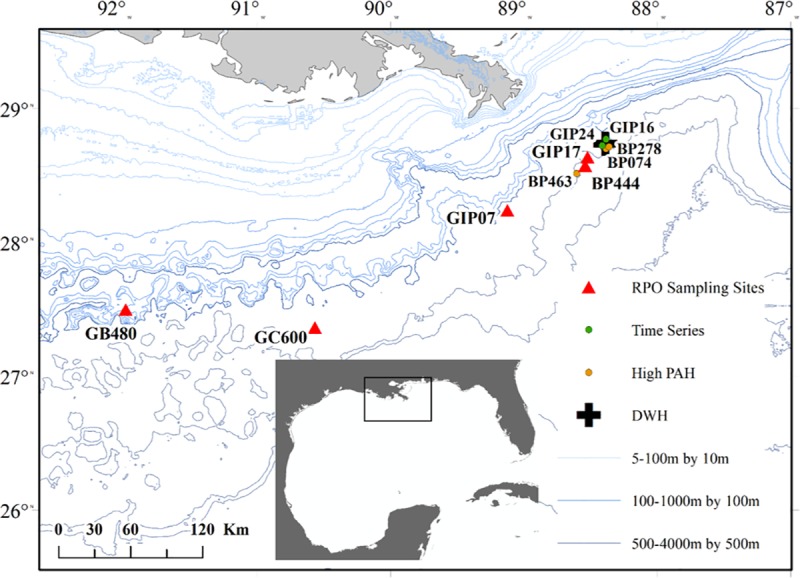
Sites of sediment collection for time series (green), high PAH (yellow), and RPO analysis (red).

Ethics statement: No permissions were required as all sites were in unprotected areas. This field study did not involve endangered or protected species. Oil spill affected sediment was collected in time series from 4 sites (GIP07, GIP17, GIP24, GIP16, Fig 1) from 2010–2017 and analyzed for bulk radiocarbon. In 2015, we also sampled 4 sites that had contained high PAHs in 2010 as reported by Mason et al. [23]. We revisited one of these sites, BP444, again in 2017. Sediment from 5 sites in the northern Gulf of Mexico (GOM) were analyzed using RPO, including 2 of the time series sites (GIP07 and GIP17), 1 high PAH site (BP444), 1 natural seep (GC600) and 1 non-hydrocarbon influenced control (GB480) site were analyzed using ramped pyrolysis/oxidation (Fig 1 and Table 1). Sediment samples were frozen upon collection, returned to the lab, thawed, treated in 10% HCl to remove carbonates, washed, freeze-dried and ground.

**Table 1:** Sites examined in this study, measurements performed, location and date.

		Latitude	Longitude	RPO and Bulk	Bulk only
GIP07	Time Series	28.2397	-89.1207	2010, 2011, 2014	2016, 2017
GIP 16	Time Series	28.7231	-88.4096	—	2010–2012, 2014
GIP17	Time Series	28.6373	-88.5188	2010, 2011, 2015	2016, 2017
GIP 24	Time Series	28.7706	-88.3812	—	2010, 2011
BP074	High PAH	28.6995	-88.3812	—	2015
BP278	High PAH	28.7150	-88.3590	—	2015
BP444	High PAH	28.575	-88.5377	2015	2017
BP463	High PAH	28.5140	-88.6005	—	2015
GC600	Seep	27.3645	-90.5629	2014	—
GB480	Control	27.4977	-90.9797	2015	—

The Ramped Pyrolysis/Oxidation System (RPO) at the National Ocean Sciences Accelerator Mass Spectrometer facility (NOSAMS) was used to serially oxidize sediments in a controlled environment following the instrument protocol for pyrolysis in Rosenheim et al. [13]. All quartz glassware used in this study was pre-combusted at 850°C for 1 hour prior to use. An aliquot of sediment, between ~80–110 mg, depending on the C content, was loaded into a pre-combusted quartz tube, between layers of pre-combusted quartz wool, and inserted into the combustion oven, sealed away from atmosphere. Macondo crude oil was added to a small quartz cup and loaded into the quartz tube. A total gas flow of 35 mL/min of helium with 8% oxygen flowed through the sample as the temperature was consistently ramped up to 800–1000°C (5°C /min). Prior to being trapped on the vacuum line, the evolved CO_2_ was measured with a Sable Systems CA-10a CO_2_ Analyzer, which was then used to plot the thermographs. The CO_2_ was integrated by cryogenically trapping using N_2_(l) over selected temperature intervals based on each sample’s unique CO_2_ evolution profile, by routing the flowing gases to different traps. Ultimately, the samples were expanded into a vacuum separations line, purified using alternating slurries of isopropanol cooled to liquid-solid phase transition with dry ice (CO_2_(s)), quantified manometrically using a capacitive diaphragm pressure gauge, and then sealed into a borosilicate glass ampoule. The samples were reduced to graphite using the hydrogen reduction method [24]. Roughly 10% of CO_2_ was diverted during the graphitization process to be analyzed for δ^13^C. The graphite was analyzed for Δ^14^C on the USAMS instrument (3MV Tandetron) at NOSAMS [25–26]. Hemingway et al. [27] estimated the contamination blank for a typical RPO analysis on this system was 3.7 ± 0.6 μg C, with δ^13^C = -29.1± 0.1‰ and potentially Δ^14^C = -449 ± 41‰. The blank carbon correction for δ^13^C ranged between -0.02 to +0.15‰ and Fm ranged from -0.002 to +0.002 (Δ^14^C ~ 3–4 ‰) [27]. Due to the small size of these corrections relative to the large differences in endmembers in this experiment, the data herein were not corrected. Bulk Δ^14^C analysis was completed at either NOSAMS or the University of Georgia Center for Applied Isotope Studies (UGA) using conventional sedimentary organic carbon ^14^C dating and graphitization approaches [24,28].

## Results and discussion

### Time series of bulk ^14^C values

**Fig 2:**
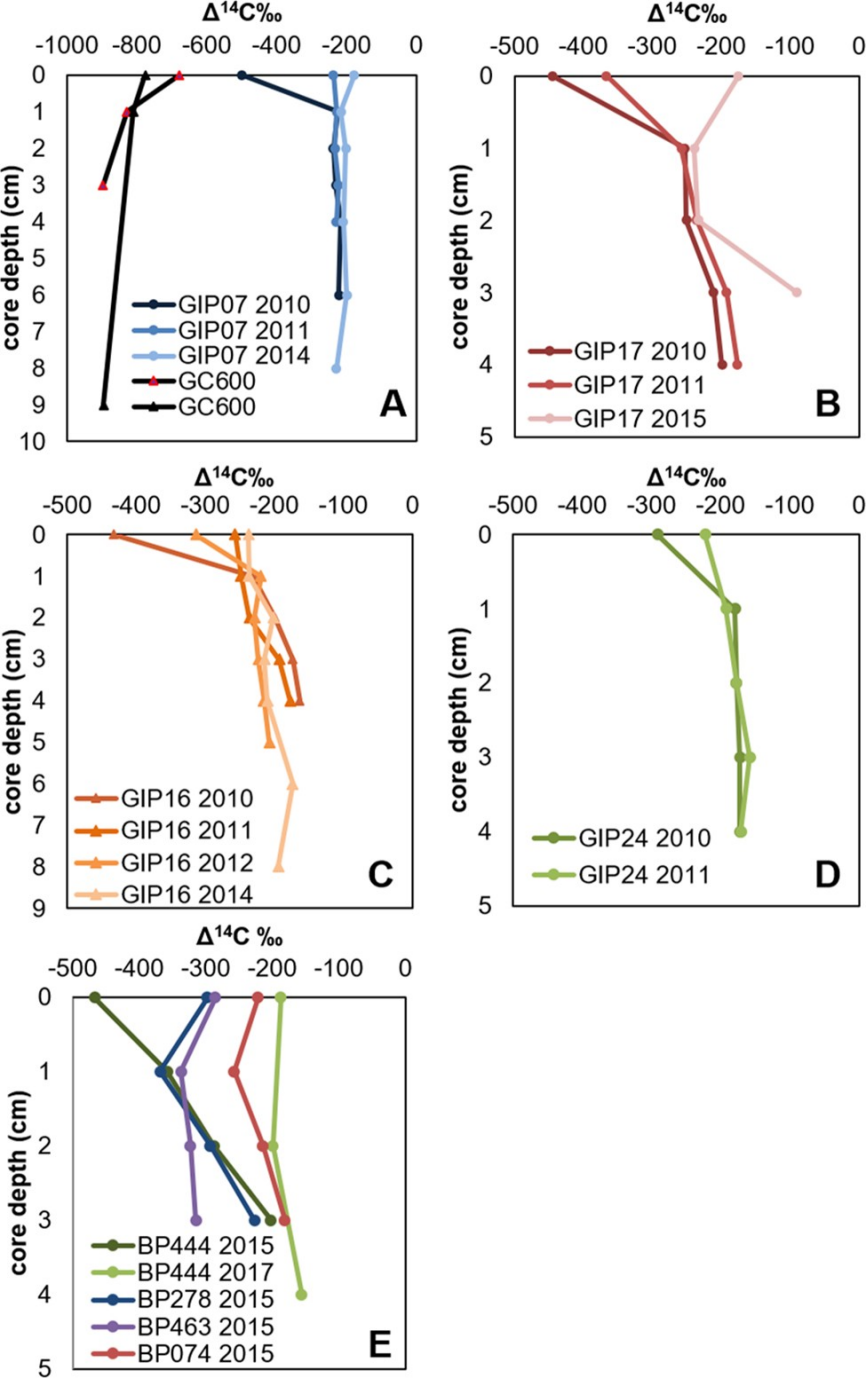
Bulk radiocarbon signatures of time series and high PAH sediment from DWH affected sites and natural seep GC600. A) GIP07 and replicate cores from mega seep site GC600, B) GIP17, C) GIP16, D) GIP24, E) high PAH sites reported by Mason et al. [23] sampled in 2015 and Site BP444 revisited in 2017. Bulk Δ^14^C values of sediment in A-D exhibit recovery back to baseline values, while the high PAH sites in E indicate that not all sites had returned to baseline-like values.

As discerned by increasing isotopic enrichment, we observed recovery of bulk radiocarbon and stable carbon isotopes in sediments collected in time series from 4 sites (Fig 2A–2D). In general, all the spill affected sites showed recovery over the sampling time period. Δ^14^C signatures were as low as ~-501‰ (representative of a mixture of 38% ^14^C-free petrocarbon and 62% background) in 2010, and over time returned towards background values which are estimated to be Δ^14^C = -200±29‰ [3]. It should be noted that oil-spill affected sites present in a fundamentally different manner from seep sites (Fig 2A). Oil spill sites contain a surface veneer of fossil carbon overlying more ^14^C enriched “younger” C; as also noted by Adhikari et al. [10], whereas seep sites have relatively uniform ^14^C depleted fossil carbon signature through all depths. In addition to the stratified nature of oil spill contaminated sites, evidence of Macondo hydrocarbons in the particulate phase in the deep-water hydrocarbon plume was found as far as 190 km southwest of the Macondo wellhead [29]. In 2015, the surface sediment Δ^14^C signatures of the 4 high PAH sites ranged from -187.1 to -467.5‰ (Fig 2E) indicating that not all sites in the northern Gulf of Mexico had fully recovered to baseline values by 2015. Interestingly, at two of the sites, the sediment below the surface layer from 1–2 cm was just as depleted if not more so than the surface with Δ^14^C signatures ranging from -257.5 to -369.1‰ (Fig 2E). Below that depth, Δ^14^C values increased. BP444 (high PAH site) was revisited again in 2017 where we observed baseline values. We further explored the isotopic recovery at these sites by using RPO to analyze the potential evolution of the sedimented petrocarbon from the time series and the high PAH sites.

### Changes in patterns of thermal stability

**Fig 3:**
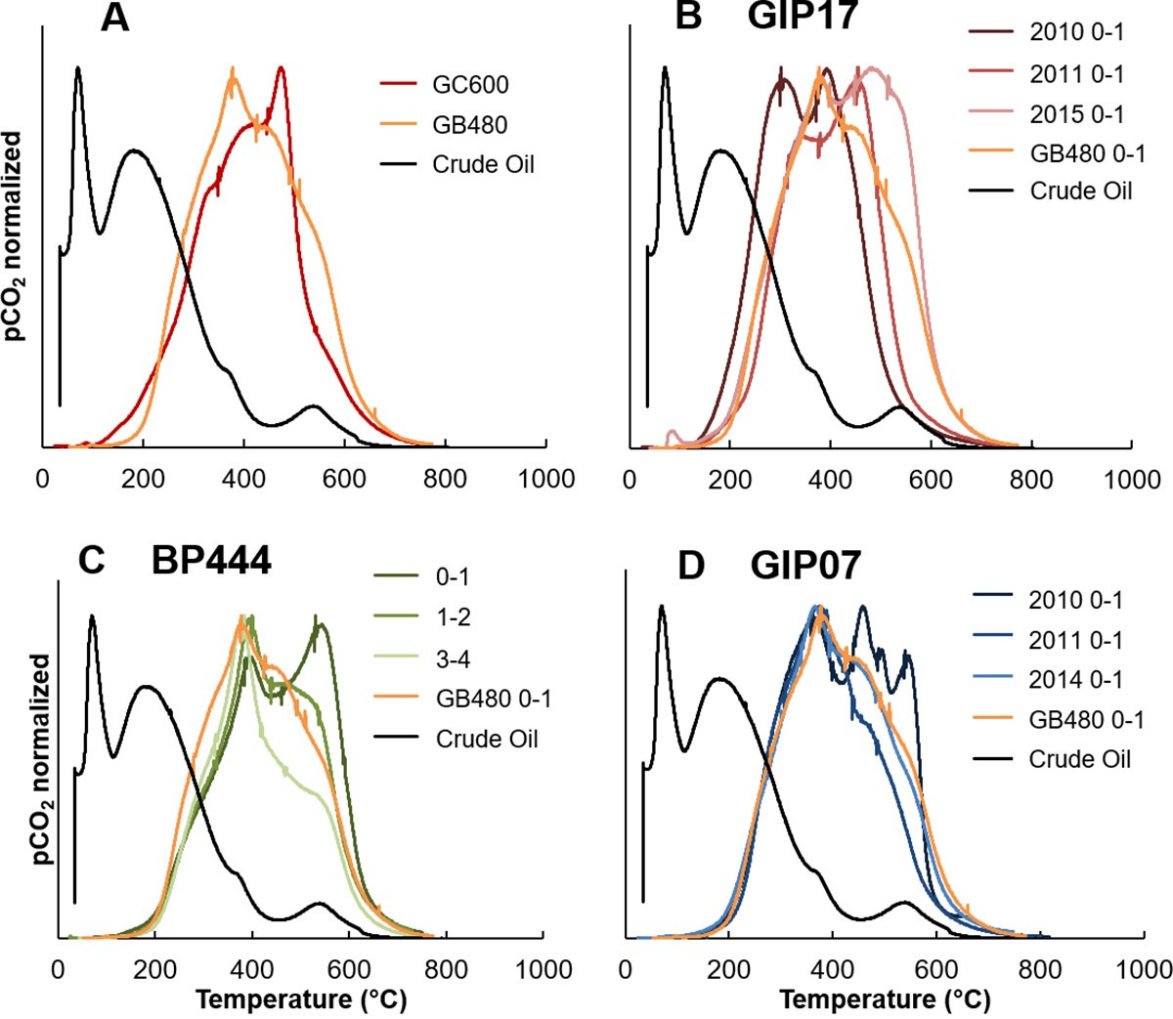
CO_2_ evolution thermographs. A) Crude Oil, Seep site GC600 and Control site GB480, B) GIP17, crude oil and control site, C) BP444, crude oil and control site, D) GIP07, crude oil and control site. Vertical “tic” marks designate temperature boundaries of isotopic sample collections.

Our second hypothesis was that the oil-residue deposited in the sediment following the oil spill would be more thermally stable than fresh Macondo oil. The contaminated sites that were run for RPO, GIP17, BP444 and GIP07, all exhibited thermal CO_2_ evolution peaks at higher temperatures than the fresh oil (Fig 3). The evolved CO_2_ thermographs from sediment, naturally oiled (seep) and non-oiled (control), were different from the crude oil thermograph, which exhibited two large low temperature peaks before ~200°C and tapered off at higher temperatures (Fig 3A). The thermograph for the seep, GC600, had two shoulders at lower temperatures, building to a peak at ~460°C, before rapidly falling off (Fig 3A). The petrocarbon present in GC600 sediment was clearly more thermochemically stable relative to the Macondo crude oil based on these thermographs. In contrast to the crude oil and sediment from GC600, sediment from the control site, GB480, exhibited a single prominent peak at ~370°C that tapered off with two more shoulders at higher temperatures (Fig 3A). CO_2_ thermographs from presumably uncontaminated sediments underlying oil-contaminated sites followed this same pattern yielding a prominent peak at ~370°C at site BP444 (3-4cm, Fig 3C), as did sediments from GIP07 in 2014 which had returned to background-like values (Fig 2). We assign this peak to typical northern Gulf sedimentary organic carbon.

Our third hypothesis was that there would be a change in CO_2_ evolution from lower temperatures to higher temperatures in the thermographs as the petrocarbon became increasingly degraded over time. We found evidence consistent with this hypothesis in the three contaminated sites, GIP17 (Fig 3B) and GIP07 (Fig 3D), and BP444 (Fig 3C). The thermograph from GIP17 2010, the oil contaminated site closest to the well head, had a lower temperature peak straddling 300°C, and exhibited the peak at ~370°C, similar to the control site. GIP17 profiles from 2011 and 2015 also exhibited the peak at ~370°C, but the peak at 300°C shifted to higher temperatures over time to 450°C in 2011 and then 480°C in 2015 (Fig 3B). The peaks at 450°C and 480°C were similar to the peak evolving at 460°C at the seep site, GC600 (Fig 3A), indicating extremely weathered petrocarbon. The CO_2_ thermograph from GIP07 2010, unlike the GIP17 curve, initially exhibited three peaks at higher temperatures, with two peaks <500°C and one >500°C (Fig 3D). CO_2_ thermographs from subsequent years at site GIP07 (2011, 2014) are similar to the control site, exhibiting the prominent peak at 370°C, and the loss of the extra mid-high temperature peaks observed in 2010 (Fig 3D). As the weathered material evolving at around 500°C would presumably be relatively un-biodegradable, we suggest that this material may have been resuspended.

**Fig 4:**
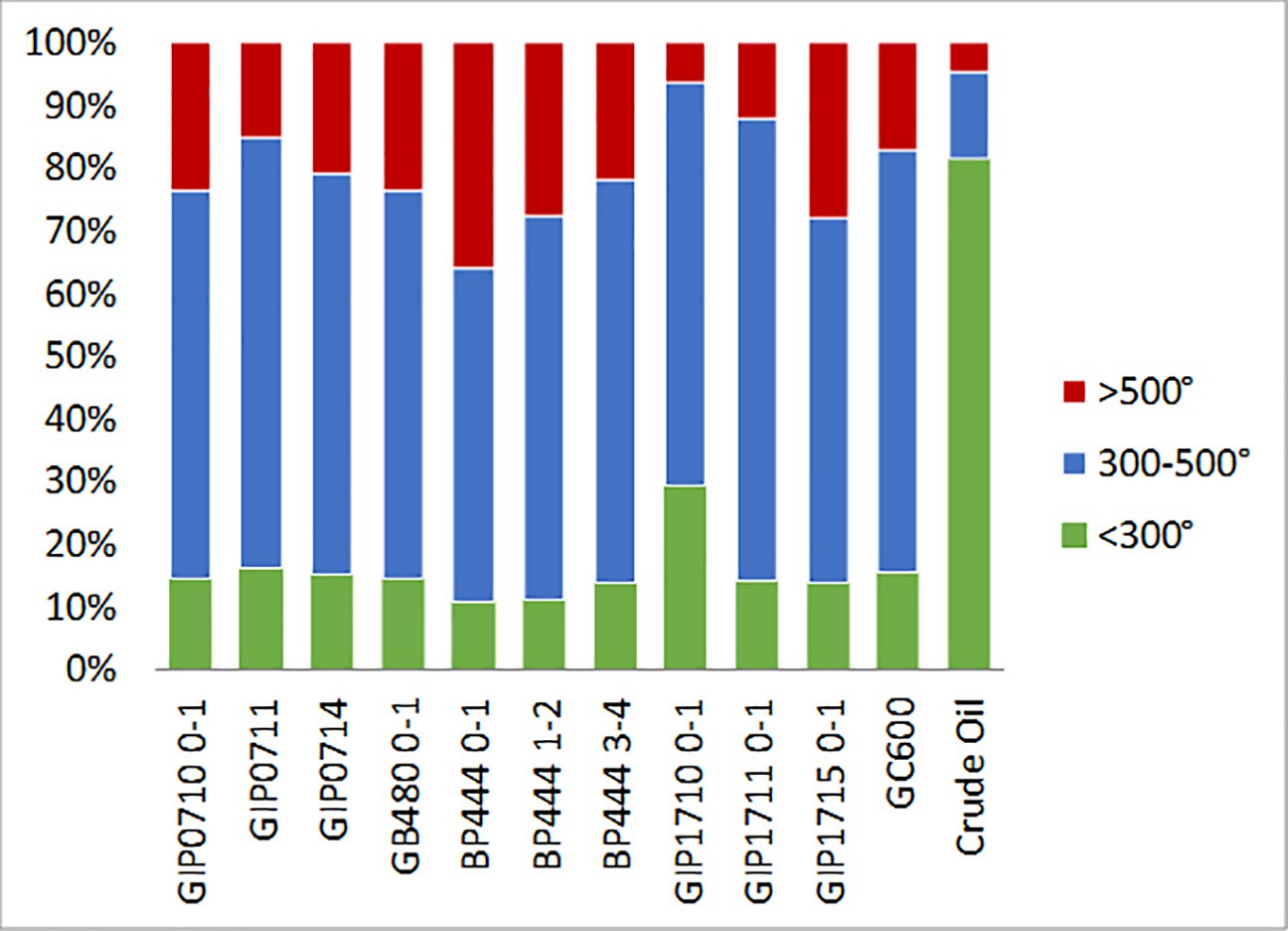
Percent CO_2_ evolved from low (300°C), medium (300–500°C) and high (>500°C) temperature.

The depth profile collected in 2015 from site BP444 was similar to GIP07 and GB480, with all depths displaying a peak at ~370°C (Fig 3C). BP444 2015 0-1cm had a secondary peak at high temperature ~530°C, which decreased to a shoulder at deeper depths within the core. Considering all the data in Fig 3, we generally observed a peak at 370°C, the control site peak. Petrocarbon evolved at temperatures below 370°C, or above it, depending upon its “maturity” or evolution towards a more recalcitrant form. Changes in the magnitude and temperature of evolution of the peaks indicate changes in the thermochemical stability of the seafloor petrocarbon as it matured from evolving at 300°C, (Fig 3B, GIP17) to over 450–500°C, (BP444, GB480 and GIP07). Pendergraft and Rosenheim [12] observed as we did, that fresh oil evolved CO_2_ at temperatures well below 300°C. They observed that over time, as the oil weathered in the nearshore sediments that they studied, it shifted towards evolution at higher and higher temperatures. Consistent with this interpretation, we observed that at GIP17, over time, and with more biodegradation the evolved CO_2_ shifted towards higher temperatures. At GIP07, we believe our sampling effort temporally missed the less weathered, lower temperature evolving petrocarbon, and that our initial sample contained more weathered petrocarbon, similar to that found at GIP17 in 2015. Note the similarity of the samples GIP07, 0–1 in 2010, and GIP17, 0–1 in 2015 (Table 2 and Fig 4). Isotopic results (below) are consistent with this interpretation.

**Table 2:** Comparison of bulk measured isotopic values vs RPO weighted average bulk values. A paired t-test indicated no difference for Δ^14^C values, p = 0.259, t = 1.103, while bulk measured Δ ^13^C values were significantly enriched relative to the RPO weighted average (p = 0.002, t = 4.158).

	Bulk Measured		Bulk Averaged		Difference	
	δ^13^C	Δ^14^C	δ^13^C	Δ^14^C	δ^13^C	Δ^14^C
GIP07 2010 0–1	-22.8	-501.5	-24.5	-447.5	1.7	-54.0
GIP07 2011 0–1	-21.7	-237.3	-23.4	-247.5	1.7	10.2
GIP07 2014 0–1	-22.2	-177.4	-23.4	-242.2	1.2	64.8
GB480 2015 0–1	-22.2	-132.4	-22.9	-189.4	0.7	57.0
BP444 2015 0–1	-22.1	-467.5	-22.8	-422.4	0.7	-45.1
BP444 2015 1–2	-21.8	-358.1	-22.4	-361.4	0.6	3.3
BP444 2015 3–4	-21.1	-202.1	-21.9	-207.0	0.9	4.9
GIP17 2010 0–1	-23.1	-445.2	-25.2	-491.6	2.1	46.4
GIP17 2011 0–1	-23.8	-368.5	-23.7	-396.1	-0.1	27.6
GIP17 2015 0–1	-23.2	-237.3	-23.2	-264.3	0.0	27.0
GC600 2014 0–1	-29.0	-915.7	-29.4	-912.6	0.4	-3.1

The percent oxidized by the temperature intervals low: <300°C, medium: 300–500°C and high: >500°C was calculated to determine shifts in the thermochemical stability of the carbon in the sediments through time [12]. These calculations were performed using the CO_2_ data continuously collected during RPO prior to purification on the vacuum line. The majority of the crude oil, 82%, was oxidized below 300°C, whereas all of the sediment, both oil-contaminated and unaffected, was primarily oxidized at temperatures above 300°C, with only 10–16% oxidized at lower temperatures (Fig 4). Sediment from GIP17 was consistent with our third hypothesis, with 2010 having the most C oxidized <300°C, 29%, decreasing over time to 14% in 2015. At GIP17, the percent oxidized at >500°C increased over time from 6% in 2010 to 28% in 2015. The down core profile for BP444 (2015) had similar percentages for C oxidized <300°C, ranging from 11–14%, while at high temperatures (>500°C) there was a decrease in percent oxidized down core from 36% at 0-1cm to 28% at 1-2cm and 22% from 3-4cm. The majority of the C was oxidized in the mid-range of temperatures (300–500°C) throughout all sampling years at all sites, summarized in Table 3.

**Table 3:** Percent of CO_2_ evolved at low, medium, and high temperatures.

	<300°C	300–500°C	>500°C
Crude oil	82%	14%	5%
GC600 2014 0–1	16%	67%	17%
GB480 2015 0–1	15%	62%	23%
GIP17 2010 0–1	29%	64%	6%
GIP17 2011 0–1	14%	74%	12%
GIP17 2015 0–1	14%	58%	28%
GIP07 2010 0–1	14%	62%	24%
GIP07 2011 0–1	16%	69%	15%
GIP07 2014 0–1	15%	64%	21%
BP444 2015 0–1	11%	53%	36%
BP444 2015 1–2	11%	61%	28%
BP444 2015 3–4	14%	65%	22%

Relative to Pendergraft et al. [11] and Pendergraft and Rosenheim [12], our thermographs were shifted towards higher temperatures, even in 2010, compared to their initial oiled marsh samples, which exhibited CO_2_ evolution at temperatures more similar to crude oil. We suggest that degradation of the hydrocarbons en route prior to deposition on the deep seafloor would cause these differences. Almost half of the hydrocarbons released from the broken well head rose to the surface, forming a thick oil slick, before sinking, potentially during a Marine Oil Snow Sedimentation and Flocculent Accumulation (MOSFFA) event [30–34]. Smaller hydrocarbon droplets (<100 μm) suspended in the water, formed a deep-sea plume that travelled southwest of the wellhead [35]. Both pools of hydrocarbons were exposed to extensive and rapid degradation while in the oxic water column from microbes, dissolution, temperature, and pressure changes [36]. Through microbial processes, hydrocarbons from the surface and the deep-water plume formed aggregates or flocculants, which caused them to sink to the seafloor [1,9,33–34]. Oil degradation was faster in the water column than it was following deposition on the seafloor [5,9]. Bagby et al. [9] modeled the potential oil degradation rates and found that the size of the oil compound and aggregated particle affected the speed of degradation in the water column and sediment; the larger the particle and compound, the slower the degradation rate. This longer degradation period prior to settling to the seafloor for the deep-water samples accounts for the differences we observe between the crude oil and DWH contaminated sites and the marsh sediment from Pendergraft and Rosenheim [12].

The difference in the degradation period could account for the differences in the thermographs for GIP17 and GIP07. The oil deposited at GIP07 (~90km from the wellhead), travelled further and therefore degraded more before settling out than the oil deposited at GIP17 (~17km from the wellhead). This extended degradation period was reflected in the thermographs by the temperature differences between the initial sampling years. The thermographs from GIP17 in 2011 and 2015 and BP444 (0–1 and 1–2) from 2015 had more CO_2_ evolved at higher temperatures, suggesting they had similar degradation experiences.

### Trends in the Δ^14^C composition of evolved CO_2_

**Fig 5:**
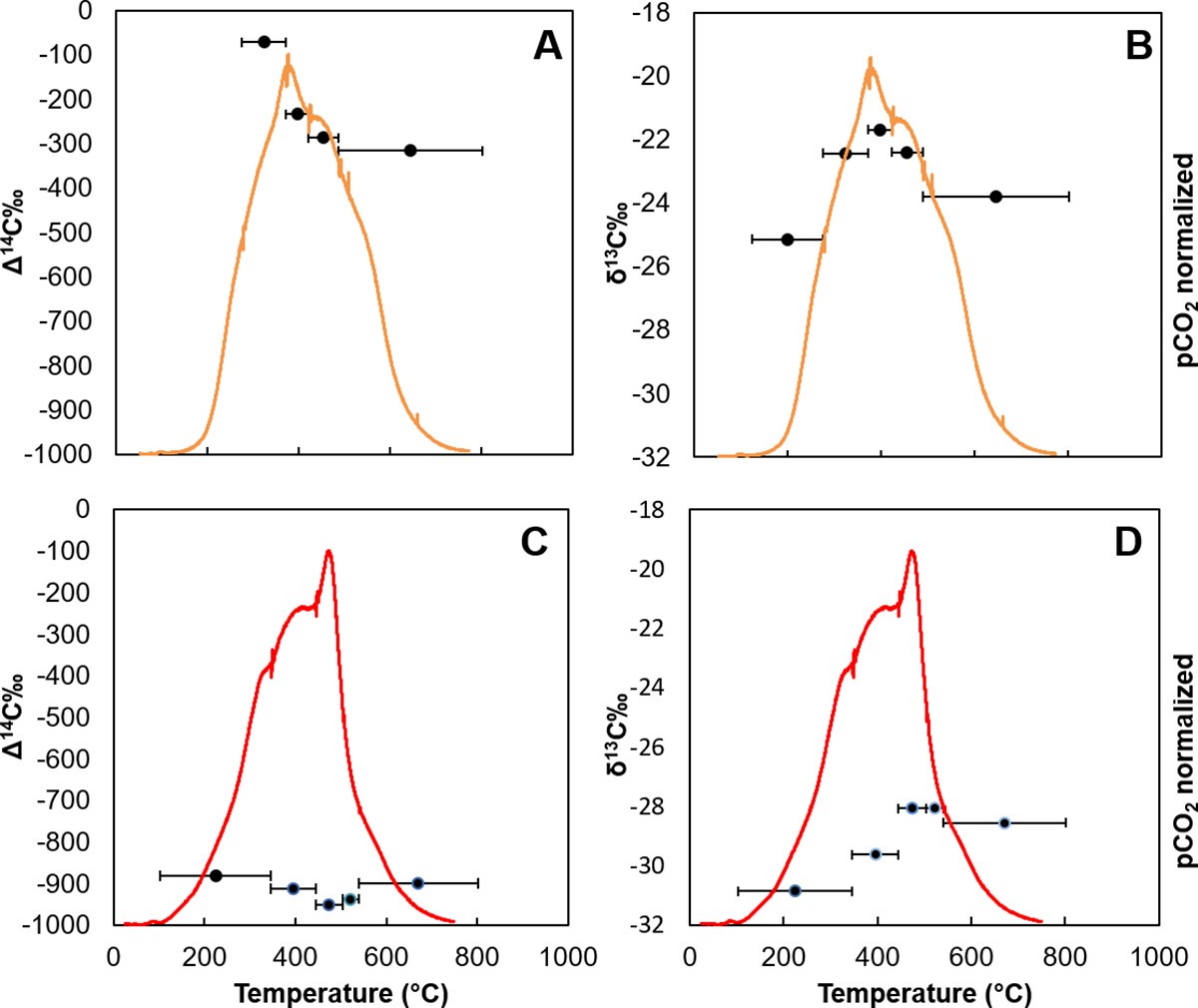
CO_2_ thermograph and isotopic composition of evolved CO_2_. Temperature interval of CO_2_ fractions indicated by horizontal bars. A) Control site GB480: Δ^14^C, B) Control site GB480 δ^13^C, C) Seep site GC600 Δ^14^C, D) Seep site GC600 δ^13^C.

We observed marked differences between the control (GB480) and seep site (GC600) due to the presence (GC600) and absence (GB480) of petrocarbon (Fig 5). Relative to the seep site, the control site had higher Δ^14^C values over all CO_2_ fractions, including CO_2_ from lower temperatures. Evolved CO_2_ fractions had decreasing Δ^14^C values as temperature increased so that the final fraction was Δ^14^C = -316.1‰ (Fig 5A, Tables 2 and 4). The seep site had consistently low values, indicating ^14^C depletion, with Δ^14^C ranging between -881.1 to -950.5‰ over all temperature fractions (Fig 5C). The δ^13^C value of the control was lowest in the first fraction, δ^13^C = -25.1‰, then increased at the ~370°C peak, δ^13^C = -21.7‰, before decreasing again in the final fraction (Fig 5B). The seep sediments followed a similar pattern, with the lowest being the first fraction, increasing at the peak and then decreasing again for the final fraction, however, the δ^13^C values of the CO_2_ evolved from the seep sediment had low δ^13^C values across all fractions, < -28‰ over all temperatures (Fig 5D).

**Table 4:** Summary of ramped pyrolysis/oxidation (RPO) results.

Accession #	Sample ID	Collection Year	δ^13^C	Δ^14^C	CO_2_ (μmol)	Start T (°C)	Stop T (°C)
**GIP0710 0–1**	**Bulk weighted average**		**-24.5**	**-447.5**	**182.4**	** **	** **
OS-132465	GIP0710 0–1 F1	2010	-23.8	-181.8	55.9	125.0	356.8
OS-132466	GIP0710 0–1 F2	2010	-22.0	-315.5	23.7	356.8	396.3
OS-132467	GIP0710 0–1 F3	2010	-23.5	-442.8	20.9	396.3	436.7
OS-132468	GIP0710 0–1 F4	2010	-25.7	-635.0	28.0	436.7	484.5
OS-132469	GIP0710 0–1 F5	2010	-26.1	-661.6	26.0	484.5	534.8
OS-132470	GIP0710 0–1 F6	2010	-26.0	-706.5	28.0	534.8	801.3
**GIP0711 0–1**	**Bulk weighted average**		**-23.4**	**-247.5**	**122.6**	** **	** **
OS-133695	GIP0711 0–1 F1	2011	-24.4	-112.6	20.1	125.0	305.5
OS-133696	GIP0711 0–1 F2	2011	-22.3	-204.0	35.6	305.5	384.8
OS-133697	GIP0711 0–1 F3	2011	-22.0	-289.6	23.7	384.8	436.3
OS-133698	GIP0711 0–1 F4	2011	-23.4	-282.6	16.9	436.3	482.8
OS-133699	GIP0711 0–1 F5	2011	-25.3	-348.9	26.3	482.8	801.6
**GIP0714 0–1**	**Bulk weighted average**		**-23.4**	**-242.2**	**158.8**	** **	** **
OS-133700	GIP0714 0–1 F1	2014	-25.3	-85.2	14.0	125.0	273.4
OS-133701	GIP0714 0–1 F2	2014	-23.4	-104.8	24.6	273.4	336.2
OS-133702	GIP0714 0–1 F3	2014	-22.2	-208.4	31.2	336.2	391.5
OS-133703	GIP0714 0–1 F4	2014	-23.0	-282.0	44.6	391.5	480.3
OS-133704	GIP0714 0–1 F5	2014	-24.0	-352.1	44.3	480.3	801.9
**GB480 0–1**	**Bulk weighted average**		**-22.9**	**-189.4**	**139.4**	** **	** **
OS-133690	GB480 0–1 F1	2015	-25.1	176.9	12.1	125.0	276.8
OS-133691	GB480 0–1 F2	2015	-22.4	-71.6	36.7	276.8	373.0
OS-133692	GB480 0–1 F3	2015	-21.7	-233.9	23.4	373.0	422.0
OS-133693	GB480 0–1 F4	2015	-22.4	-287.2	28.3	422.0	488.7
OS-133694	GB480 0–1 F5	2015	-23.8	-316.1	38.9	488.7	802.4
**BP444 0–1**	**Bulk weighted average**		**-22.8**	**-422.4**	**95.9**	** **	** **
OS-132471	BP444 0–1 F1	2015	-22.7	-215.5	26.7	140.0	381.3
OS-132472	BP444 0–1 F2	2015	-21.7	-379.8	22.5	381.3	458.6
OS-132473	BP444 0–1 F3	2015	-23.2	-482.1	20.1	458.6	526.4
OS-132474	BP444 0–1 F4	2015	-23.7	-619.3	18.5	526.4	586.2
OS-132434	BP444 0–1 F5	2015	-22.8	-624.5	8.1	586.2	760.6
**BP444 1–2**	**Bulk weighted average**		**-22.4**	**-361.4**	**101.6**	** **	** **
OS-133821	BP444 1–2 F1	2015	-23.5	-205.8	15.4	126.0	325.3
OS-133822	BP444 1–2 F2	2015	-21.9	-292.5	22.1	325.3	396.4
OS-133823	BP444 1–2 F3	2015	-21.4	-399.1	14.7	396.4	437.0
OS-133824	BP444 1–2 F4	2015	-22.4	-408.8	30.9	437.0	534.9
OS-133825	BP444 1–2 F5	2015	-23.1	-464.1	18.5	534.9	801.6
**BP444 3–4**	**Bulk weighted average**		**-21.9**	**-207.0**	**122.2**	** **	** **
OS-133816	BP444 3–4 F1	2015	-23.3	-96.7	21.9	125.0	321.0
OS-133817	BP444 3–4 F2	2015	-21.4	-179.2	25.5	321.0	378.8
OS-133818	BP444 3–4 F3	2015	-20.7	-257.9	18.2	378.8	415.5
OS-133819	BP444 3–4 F4	2015	-21.2	-199.9	17.7	415.5	465.6
OS-133820	BP444 3–4 F5	2015	-22.5	-267.0	38.8	465.6	801.6
**GIP1710 0–1**	**Bulk weighted average**		**-25.2**	**-491.6**	**161.5**	** **	** **
OS-132475	GIP1710 0–1 F1	2010	-25.0	-415.2	42.8	123.0	297.4
OS-132476	GIP1710 0–1 F2	2010	-25.7	-402.0	42.1	297.4	368.7
OS-132477	GIP1710 0–1 F3	2010	-24.2	-504.7	29.3	368.7	415.2
OS-132478	GIP1710 0–1 F4	2010	-26.0	-628.1	20.8	415.2	453.7
OS-132479	GIP1710 0–1 F5	2010	-25.5	-635.6	26.5	453.7	801.3
**GIP1711 0–1**	**Bulk weighted average**		**-23.7**	**-365.5**	**168.0**	** **	** **
OS-135128	GIP1711 0–1 F1	2011		-215.7	28.5	125.0	312.4
OS-135129	GIP1711 0–1 F2	2011	-22.7	-293.1	36.3	312.4	374.6
OS-135133	GIP1711 0–1 F3	2011	-23.6	-390.3	49.7	374.6	450.4
OS-135134	GIP1711 0–1 F4	2011	-24.1	-458.0	27.0	450.4	489.5
OS-135135	GIP1711 0–1 F5	2011	-24.7	-485.6	26.4	489.5	802.6
**GIP1715 0–1**	**Bulk weighted average**		**-23.2**	**-264.3**	**158.4**	** **	** **
OS-135136	GIP1715 0–1 F1	2015	-24.2	115.1	22.9	75.0	304.5
OS-135137	GIP1715 0–1 F2	2015	-22.4	-164.0	36.1	304.5	394.1
OS-135138	GIP1715 0–1 F3	2015	-22.1	-311.5	24.9	394.1	444.8
OS-135139	GIP1715 0–1 F4	2015	-23.3	-402.9	33.7	444.8	511.6
OS-135140	GIP1715 0–1 F5	2015	-24.1	-423.2	40.8	511.6	801.6
**GC600 0–1**	**Bulk weighted average**		**-29.4**	**-912.6**	**176.0**	** **	** **
OS-135141	GC600 0–1 F1	2014	-30.9	-881.1	47.0	103.0	346.3
OS-135144	GC600 0–1 F2	2014	-29.6	-911.6	59.7	346.3	444.2
OS-135156	GC600 0–1 F3	2014	-28.1	-950.5	39.3	444.2	503.9
OS-135142	GC600 0–1 F4	2014	-28.1	-936.9	12.5	503.9	539.4
OS-135143	GC600 0–1 F5	2014	-28.6	-898.4	17.5	539.4	802.2

**Fig 6:**
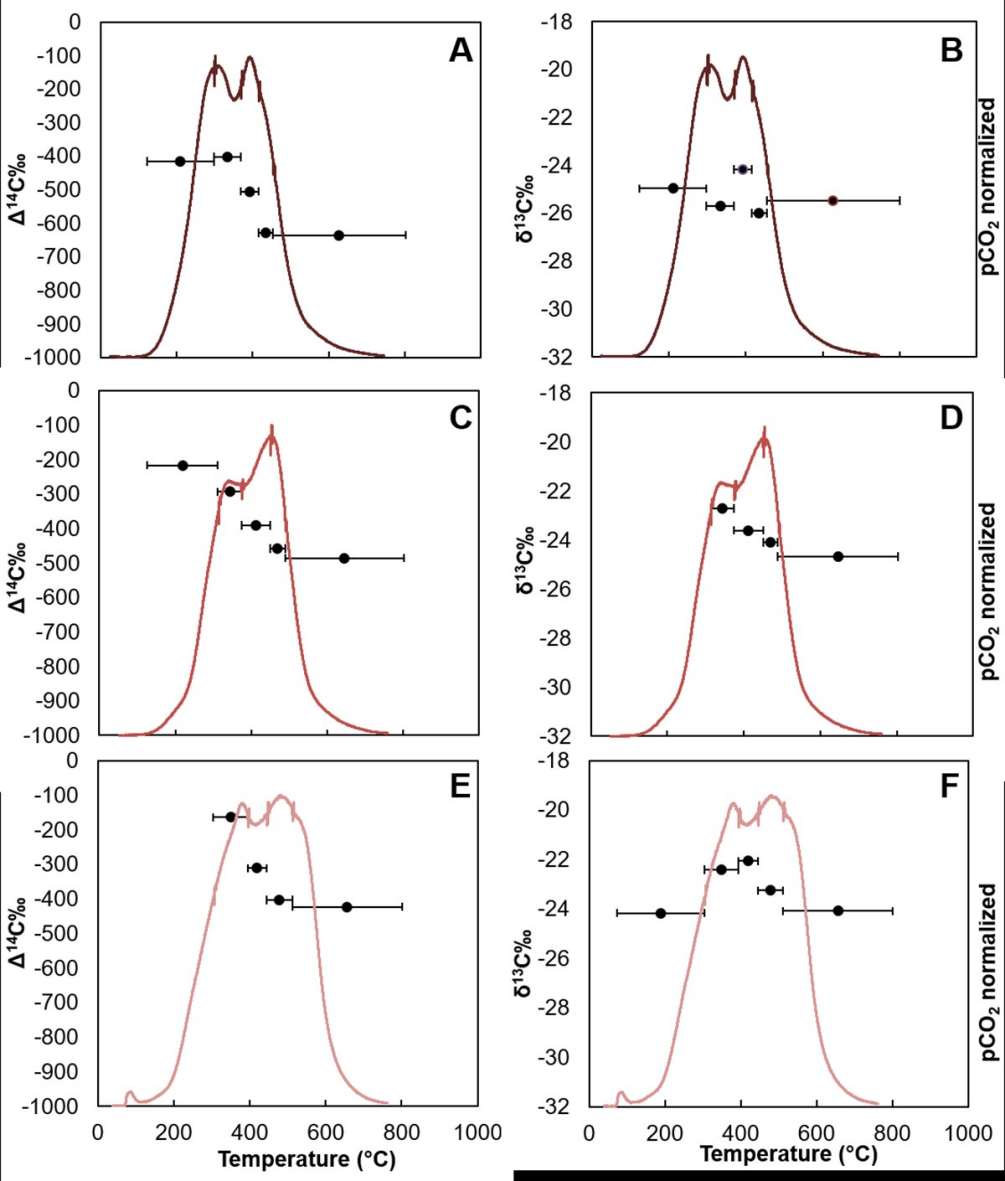
CO_2_ thermograph and isotopic composition of evolved CO_2_ for site GIP 17. Temperature interval of CO_2_ fractions indicated by horizontal bars. A) GIP17 2010 Δ^14^C, B) GIP17 2010 δ^13^C, C) GIP17 2011 Δ^14^C, D) GIP17 2011 δ^13^C, E) GIP17 2015 Δ^14^C, F) GIP17 2015 δ^13^C.

We expected the Δ^14^C value of the evolved CO_2_ at the time series sites (Fig 6) to initially exhibit lower Δ^14^C values due to petrocarbon contamination and to increase as petrocarbon degraded or was mobilized from the system by resuspension [37]. However, at these sites, typically the first fraction that evolved at the lowest temperatures was the most enriched and the last fraction was the most depleted, similar to the control, in terms of Δ^14^C (Fig 6 and Table 4). Only two samples deviated from these trends: GIP17 2010, and GC600, where all temperature fractions were highly depleted in radiocarbon. Overall, Δ^14^C values of evolved CO_2_ fractions of sediment from GIP17 became increasingly enriched over time from 2010 to 2011, and then to 2015 (Fig 6A, 6C and 6E; Tables 2 and 4).

**Fig 7:**
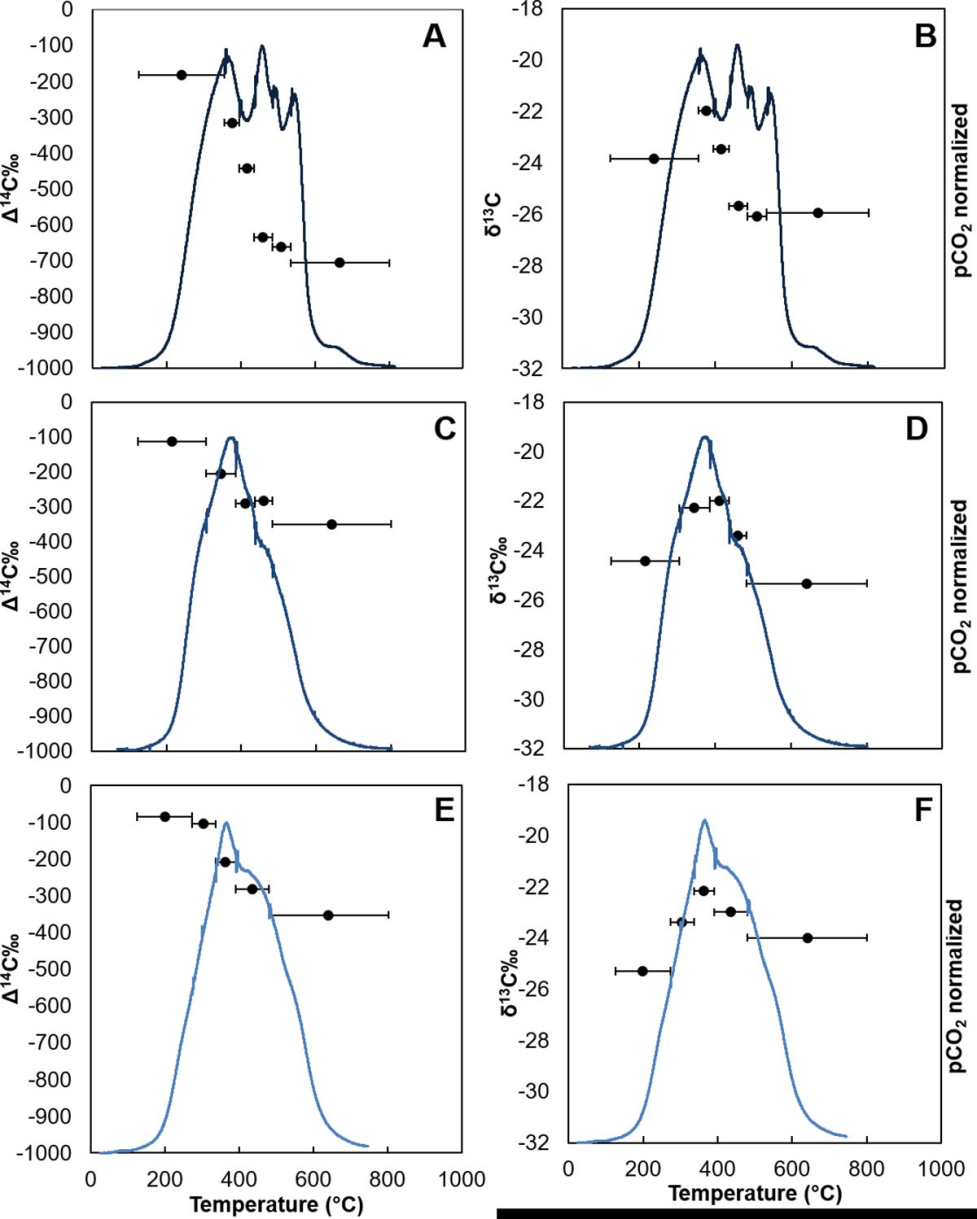
CO_2_ thermograph and isotopic composition of evolved CO_2_ for site GIP07. Temperature interval of CO_2_ fractions indicated by horizontal bars. A) GIP07 2010 Δ^14^C, B) GIP07 2010 δ^13^C, C) GIP07 2011 Δ^14^C, D) GIP07 2011 δ^13^C, E) GIP07 2014 Δ^14^C, F) GIP07 2014 δ^13^C.

**Fig 8:**
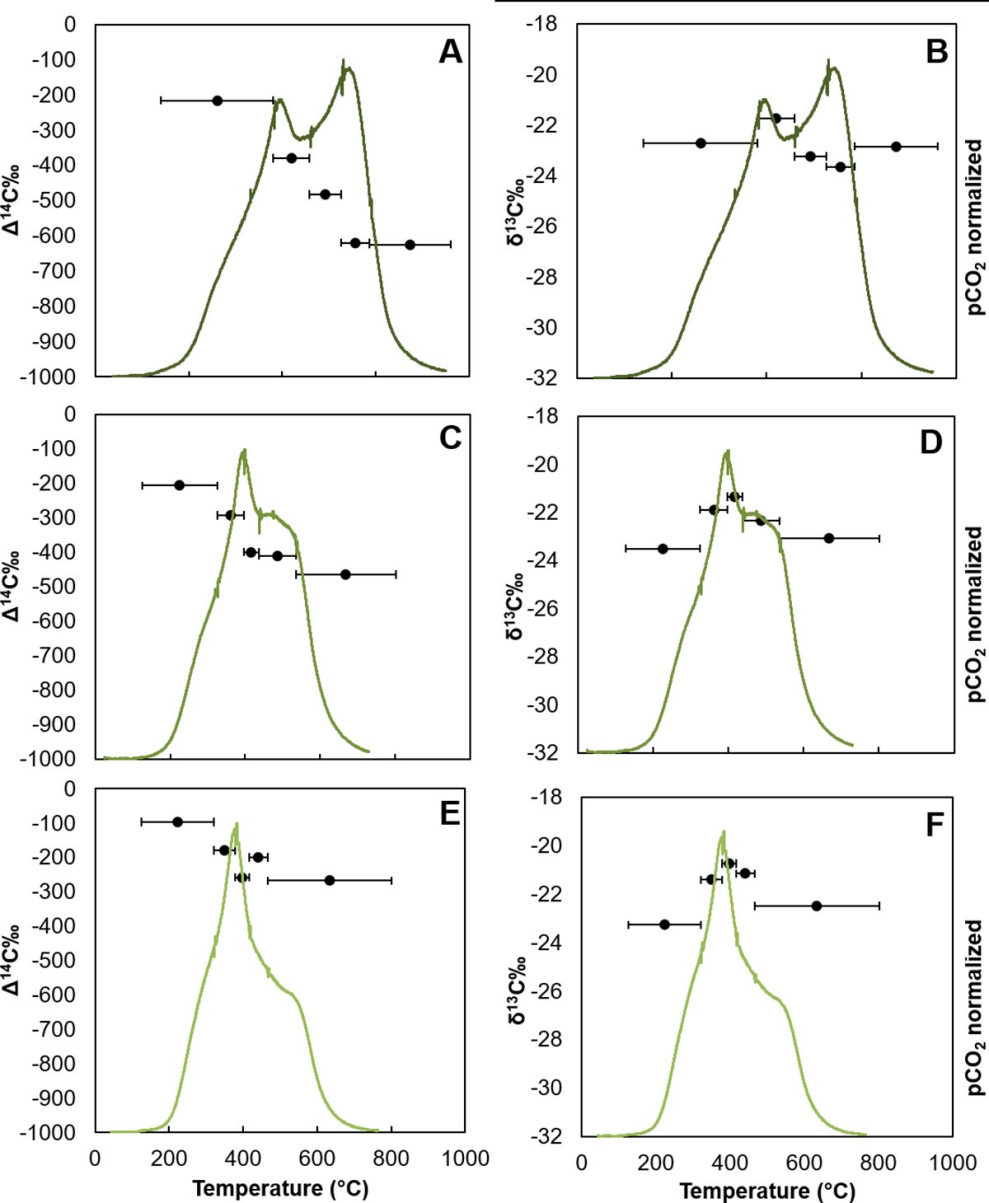
CO_2_ thermograph and isotopic composition of evolved CO_2_ for high PAH site BP444. Temperature interval of CO_2_ fractions indicated by horizontal bars. A) 0-1cm Δ^14^C, B) 0-1cm δ^13^C, C) 1-2cm Δ^14^C, D) 1-2cm δ^13^C, E) 3-4cm Δ^14^C, and F) 3-4cm δ^13^C.

The evolved CO_2_ from GIP07 also exhibited the trend of decreasing Δ^14^C signatures as temperature increased (Fig 7A, 7C and 7E; Table 4). At site BP444 (Fig 8), segment 0-1cm and 1-2cm had similar Δ^14^C values at lower temperatures, Δ^14^C = -215.5 and -205.8‰, but at higher temperatures, the 0-1cm segment was lower than at 1-2cm, with Δ^14^C = -624.5 and -464.1‰. Moving further down core at site BP444, the evolved CO_2_ fractions from sediment collected from 3-4cm had higher radiocarbon values than the first two segments from 0–1 and 1-2cm, with the initial and final fractions Δ^14^C = -96.7 and -267.0‰ (Fig 8A, 8C and 8E). Two samples, GB480 and GIP17 2015 0–1 cm (Table 4) exhibited initial CO_2_ evolution with high Δ^14^C and low δ^13^C values, suggesting the deposition of bomb radiocarbon that had been sequestered in the terrestrial environment, eroded, and re-deposited in the Gulf sediments.

Both the level of contamination and the distance from the source played a role in the recovery rate of an oil-contaminated site [5,9]. As mentioned previously, higher contamination may slow overall degradation rates, potentially due to larger particle sizes, which sink faster from the water column [9]. Valentine et al. [1] found consistently high levels of hopane (> 75ng/g) within 40 km of the wellhead. Similarly, Adhikari et al. [10] found elevated levels of PAHs < 35km from the well head 3 years after the blowout, whereas sediment beyond this distance returned to background levels. Because the bulk of the oil degradation occurs in the water column prior to sedimentation, the further the oil travelled, the more it degraded [4]. This supports our interpretation of the differences between the thermographs of GIP07 and GIP17. GIP07, ~90km from the well head, has three more CO_2_ peaks evolved at higher temperatures in 2010 than GIP17, which is closer to the well head, ~16.9km away. This is also reflected in the depleted C isotopes of the first fractions of evolved CO_2_ from GIP07 and GIP17.

### Trends in the δ ^13^C composition of evolved CO_2_

**Fig 9:**
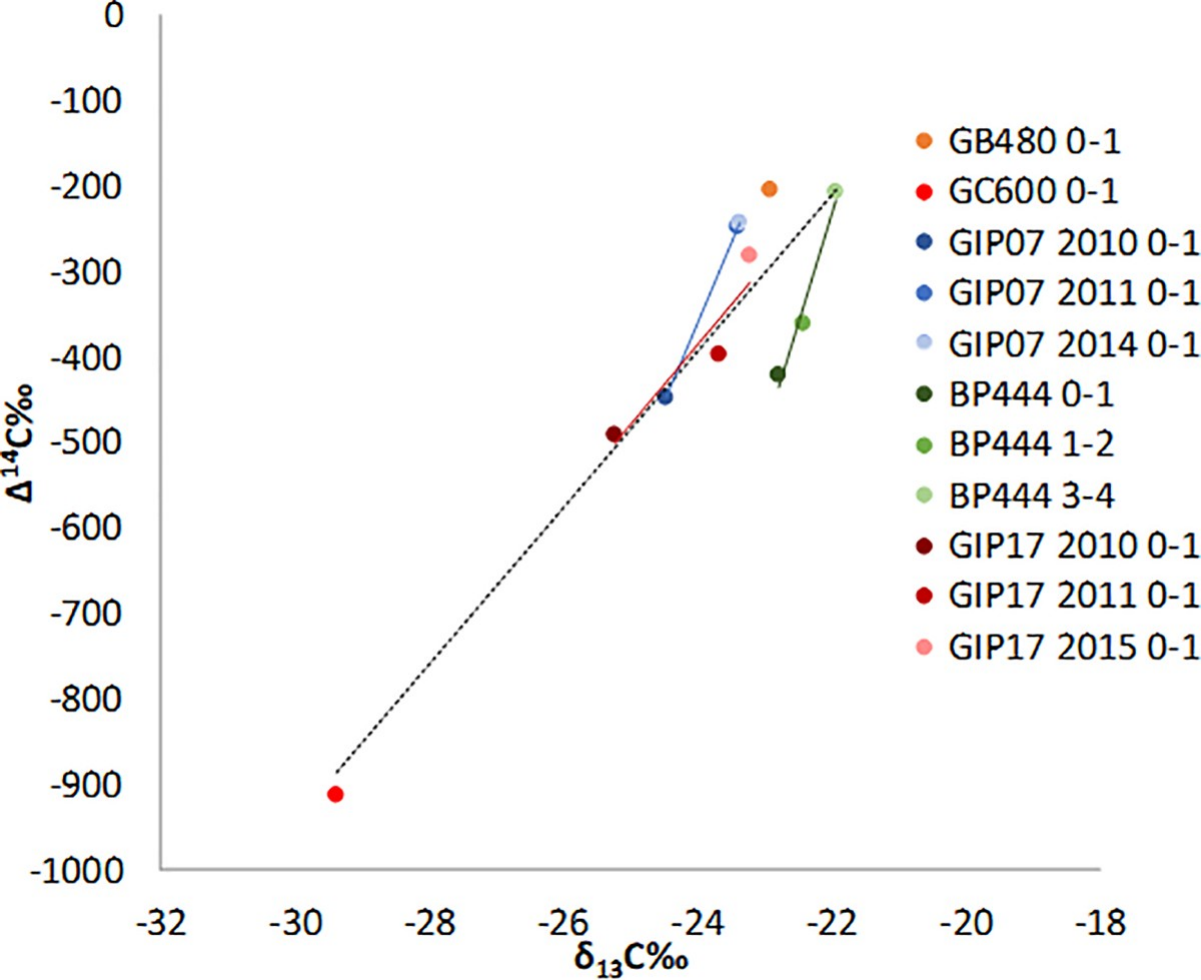
Plot of RPO averaged Δ^14^C and δ^13^C from each site. Darker shades are more contaminated, shifting towards lighter shades of the recovered time points. Dash) Overall regression (y = 92.295x + 1824.9, r = 0.9187, n = 11, p < 0.0001), time series sediments: Red) GIP17 (y = 100.06x + 2021.5, r = 0.9217, n = 3, p = 0.0783), Blue) GIP07 (y = 184.68x + 4072.6, r = .9999, n = 3, p < 0.001), Aqua) depth trend from BP444 (y = 257.42x + 5431.4, r = 0.9843, n = 3, p = 0.0157).

The trends in the δ^13^C values were more variable than the trends seen in the Δ^14^C signatures. Many of the δ^13^C signatures of the sediments followed the general trend of increasing from the lower δ^13^C value of the first fraction, and then decreasing again at higher temperatures. The peak seen at ~370°C in the control and several other samples was often the highest δ^13^C value of all the fractions (e.g., -21.7 to -22.5; Figs 5B, 6B, 6D, 6F, 7D, 7F, 8B, 8D and 8F), suggesting its origin as marine primary production, the dominant input term for sedimenting particles [38]. Overall, the bulk mean calculated from all the fractions from the 2010 sediments from GIP17 and GIP07 were the most depleted in δ^13^C, becoming more enriched in the following years (Fig 9 and Table 2). Sediments from BP444 had the highest δ^13^C values, varying by ~1.1‰ throughout the core, while δ^13^C signatures for GC600 were lower than all the other sediment. The stable carbon and radiocarbon isotope signatures for all temperature fractions are summarized in Table 4.

Several studies have explored the potential of anoxic biodegradation of oil causing enrichment in δ^13^C of the remaining reservoir. Wilkes et al. [39] incubated alkylbenzene utilizing sulfate-reducing bacteria in oil amendments and analyzed the specific oil compounds at the beginning and ending of the experiment. Wilkes et al. [39] found that as more of a compound was degraded, the heavier the remaining compound reservoir became. Griebler et al. [40] found similar results to Wilkes’ in an anoxic fresh water oil contaminated site, with specific compounds showing carbon isotope enrichment of the remaining reservoir of specific oil compounds. Sun et al. [41] found similar increases of the δ^13^C of low molecular weight n-alkanes, up to 4‰, during heavy biodegradation, but found no fractionation in high molecular weight compounds even during heavy biodegradation. This resulted in no change to the bulk δ^13^C signatures of the oil, no matter the level of degradation. Whereas these studies were in anaerobic, closed systems, the fractionation of δ^13^C during biodegradation of oil, in combination with the mixing of modern surface production carbon, could account for the increase we observe in the δ^13^C of the evolved CO_2_ of sedimented petrocarbon, the residual of the oil released from the oil spill. The petrocarbon deposited in the GOM was further degraded than the oil reservoirs Sun et al. [41] studied. Degradation of organic matter also causes increases in δ^13^C values in terrestrial soil systems. Wynn [42] synthesizes the results from several studies of well-drained tropical soils where primarily C-3 vegetation derived organic matter accumulated and degraded down core. The degradation down core caused δ^13^C signatures of the remaining organic matter to increase, up to ~ +6‰ [43–45].

### Sediment source composition and variation

We calculated the weighted average for δ^13^C and Δ^14^C per sample, combining the mass-weighted results of the individual RPO CO_2_ fractions; which were not significantly different from the measured bulk signatures for **Δ** ^14^C (Table 2). From the RPO average we employed a two-endmember mixing model using the **Δ** ^14^C results to calculate the percent carbon from modern and fossil sources (Table 5). For modern surface sediments we used a value of Δ^14^C = ~-200±29‰ [3], and for petrocarbon we used a value of -1000‰

$${\%}_{Modern}=(|\Delta^{14}C_{petrocarbon}|+\Delta^{14}C_{Bulk\;RPO})/(|\Delta^{14}C_{petrocarbon}|‑\Delta^{14}C_{background})*100$$ Eq 1

The results of a two-endmember mixing model followed the trends of the isotopes, with the proportional contribution from petrocarbon decreasing over time as the isotope signatures increased. Our seep and control sites were at opposite ends of the spectrum with the percent C from modern sources ranging from 11%, at GC600, to 101% at GB480, while petrocarbon ranged from -1%, from GB480, to 89% at GC600. GB480 is slightly more enriched than our estimated background Δ^14^C ~ -200±29‰, which caused it to have over 100% modern sources and below 0% petrocarbon (e.g. 101% and -1%). The time series sites GIP17 and GIP07 contain 36 and 31%, petrocarbon in 2010 and decreased to 8% and 5% by 2015 and 2014. The two-endmember mixing model data are summarized in Table 5. To test for sensitivity, we varied the radiocarbon background by ±29‰ and re-analyzed three sediments from a range of signatures including: background (BP444 2015 3-4cm), mid-range (GIP17 2010 0–1), and radiocarbon depleted (GC600). The sediment closest to background had the most potential variation between 3–4%, while the mid-range varied 2–3%, and finally there was no difference in the highly depleted sediment (Table 6).

**Table 5:** Estimated percent petrocarbon from RPO analyzed sediments using a ^14^C mass balance with 2 endmembers, petrocarbon at -1000‰ and background at -200‰.

		δ^13^C	Δ^14^C	Percent Modern	Percent Petrocarbon
GB480 2015 0–1		-22.9	-189.4	101	-1
GIP17 2010 0–1		-25.2	-491.6	64	36
GIP17 2011 0–1		-23.7	-365.5	79	21
GIP17 2015 0–1		-23.2	-264.3	92	8
GIP07 2010 0–1		-24.5	-447.5	69	31
GIP07 2011 0–1		-23.4	-247.5	94	6
GIP07 2014 0–1		-23.4	-242.2	95	5
BP444 2015 0–1		-22.8	-422.4	72	28
BP444 2015 1–2		-22.4	-361.4	80	20
BP444 2015 3–4		-21.9	-207.0	99	1
GC600 2014 0–1		-29.4	-912.6	11	89

**Table 6:** Sensitivity test for 2 end member model estimating percent carbon sources.

					Adjusted Background					
		Estimated			-229‰		-200‰		-171‰	
	Δ^14^C ‰	Modern	Petro-carbon		Modern	Petro-carbon	Modern	Petro-carbon	Modern	Petro-carbon
BP444 3–4	-207.0	99%	1%		103%	-3%	99%	1%	96%	4%
GIP17 2010	-491.6	64%	36%		66%	34%	64%	36%	61%	39%
GC600 2014	-912.6	11%	89%		11%	89%	11%	89%	11%	89%

The co-variation of the RPO averaged δ^13^C and Δ^14^C was consistent with the C isotope depletion we observed due to the addition of petrocarbon (Fig 9 and Table 2). This co-variation has also been seen for particulate organic carbon and plankton [46, 47]. We estimated recovery rates as defined as increasing isotopic enrichment over time from the linear regressions calculated from the co-variation of the averaged RPO values of δ^13^C and Δ^14^C for the 0–1 cm interval at the GIP17 and GIP07 sites. Given the low sample number at each site, there was no statistical significance for one of the regressions (GIP17, closer to the wellhead), but we used it to estimate what the recovery rates might be. Of the two time series sites, GIP07, ~90km from the well head had a faster recovery rate at Δ^14^C = 46‰ per year (184.68 in 4 years) than GIP17, ~23km away, with Δ^14^C = 18.3‰ per year (91.6 in 5 years) (Fig 9). BP444 exhibited increasing Δ^14^C values with depth, becoming Δ^14^C = 257‰ less depleted over the 4cm of sediment we analyzed. The radiocarbon profile from BP444 showed a distinct depleted layer from 0-2cm overlaying more enriched sediment from 2-4cm. These noticeable layers indicated that there was little to no mixing or bioturbation from initial deposition of petrocarbon to sample collection in 2015.

Adhikari et al. [10] also analyzed DWH affected sediments using RPO and found depleted radiocarbon signatures in the higher temperature CO_2_ fractions. Additionally, they found elevated levels of PAHs near the Macondo wellhead after collection in 2013. Adhikari et al. [10] and Bagby et al. [9] found that in highly contaminated areas, their respective tracers, PAHs and hopane, persisted for 3–4 years following the blowout. These estimates are similar to our recovery estimates for sites further from the wellhead, which was **~**4 years. We found that sites closer to the wellhead, with potentially higher levels of initial contamination, took 5–6 years to reach background Δ^14^C signatures.

The slower recovery rates at BP444 could be caused by the highly variable sedimentation rates across the region during and shortly after the blowout. There was increased sedimentation in the Fall of 2010 through early 2011 corresponding to the MOSSFA event following the blowout [2], with sedimentation rates ranging between 0.48 to 2.40 g/cm^2^/year during the MOSSFA event but returning to pre-spill fluxes of 0.05 to 0.16 g/cm^2^/year later in 2011 [48]. The large spatial heterogeneity of sedimentation [2,33], could have created areas of higher contamination, which would be indicated by lower radiocarbon signatures. Even though GIP17 is closer to the wellhead by ~6km, surface sediment from GIP17 in 2015 was more enriched with Δ^14^C = -264‰, than surface sediment from BP444 in 2015, which had Δ^14^C = -422‰. Higher contamination levels would have slowed degradation rates, explaining the lower Δ^14^C signatures and slower recovery at BP444 in 2015 [1,9,49]. The massive flux of hydrocarbon contaminated material to the seafloor also reduced the size of the benthic community as well as its diversity [49,50]. With the decline in these communities, there was a reduction in the amount of bioturbation in the surface sediment, which was reflected in ^234^Th results [2]. The reduced mixing would also lead to slower Δ^14^C recovery times at site BP444.

An additional consequence of our study is to shed light on the origin of the sedimentary organic matter to the Gulf of Mexico. Gordon and Goñi [51–53] hypothesized that organic matter characterized as low-lignin, with high δ^13^C and low Δ^14^C values contributed to organic matter deposited in deep water of the northern GOM. They suggested old, highly degraded soil organic matter from historic C-4 prairie grasses along the Mississippi River as a potential source of this organic carbon. We find no evidence to support this hypothesis. The δ^13^C and Δ^14^C values from the CO_2_ thermograph of the control sediment (GB480, 0–1), closely resembles the samples from the deeper sediment at BP444, 3–4 cm, and the GIP07 2014 and we suggest that these three samples are representative of typical Gulf sediments. They all exhibited decreasing δ^13^C and **Δ** ^14^C values with increasing temperature, contrary to what would have been observed if recalcitrant C-4 organic matter was present. Recalcitrant C-4 organic matter would evolve CO_2_ with low Δ^14^C and high δ^13^C values at higher temperatures. The final temperature fraction of sediment from these samples were similar to or more depleted than marine organic matter δ^13^C ~ -20‰ and did not indicate mixing with a C-4 source with a δ^13^C ~ -14‰. The primary source of organic matter to deep water Gulf sediments appear to be marine [38].

## Conclusions

Ramped Pyrolysis/Oxidation combined with isotopic analysis of the evolved CO_2_ fractions provides valuable insight into petroleum degradation over time. Hydrocarbons deposited on the seafloor of the deep-water Gulf of Mexico took years to dissipate. Compounds of low thermochemical stability were transformed to compounds of higher thermal stability, consistent with the shift from hydrocarbon to petrocarbon. The time frame of this evolution appears to depend upon distance from the well head and the distance the oil traveled prior to deposition.

## References

[1] Valentine DL, Fisher GB, Bagby SC, Nelson RK, Reddy CM, Sylva SP, et al Fallout plume of submerged oil from Deepwater Horizon. Proc Natl Acad Sci 2014; 111(45), 15906–15911. 10.1073/pnas.1414873111 25349409 PMC4234598

[2] Brooks GR, Larson R a., Schwing PT, Romero I, Moore C, Reichart GJ, et al Sedimentation pulse in the NE Gulf of Mexico following the 2010 DWH blowout. PLoS One. 2015;10(7):1–24. 10.1371/journal.pone.0132341 26172639 PMC4501746

[3] Chanton J, Zhao T, Rosenheim BE, Joye S, Bosman S, Brunner C, et al Using natural abundance radiocarbon to trace the flux of petrocarbon to the seafloor following the Deepwater Horizon oil spill. Environ Sci Technol, 2015; 49(2), 847–854. 10.1021/es5046524 25494527

[4] Stout SA, Rouhani S, Liu B, Oehrig J, Ricker RW, Baker G, et al Assessing the footprint and volume of oil deposited in deep-sea sediments following the Deepwater Horizon oil spill. Mar Pollut Bull 2016; 10.1016/j.marpolbul.2016.09.046 27677393

[5] Stout SA, Payne JR. Macondo oil in deep-sea sediments: Part 1: sub-sea weathering of oil deposited on the seafloor. Mar Pollut Bull. 2016;111(1–2):365–80. 10.1016/j.marpolbul.2016.07.036 27488960

[6] Romero IC, Toro-Farmer G, Diercks AR, Schwing P, Muller-Karger F, Murawski S, et al Large-scale deposition of weathered oil in the Gulf of Mexico following a deep-water oil spill. Environ Pollut. 2017; 228:179–89. 10.1016/j.envpol.2017.05.019 28535489

[7] Brakstad OG, Lewis A, Beegle-Krause CJ. A critical review of marine snow in the context of oil spills and oil spill dispersant treatment with focus on the Deepwater Horizon oil spill. Mar Pollut Bull, 2018; 135, 346–356. 10.1016/j.marpolbul.2018.07.028 30301046

[8] Passow U, Ziervogel K. Marine Snow Sedimented Oil Released During the Deepwater Horizon Spill. Oceanography. 2016; 29(3):118–25. 10.5670/oceanog.2016.76

[9] Bagby SC, Reddy CM, Aeppli C, Fisher GB, Valentine DL. Persistence and biodegradation of oil at the ocean floor following *Deepwater Horizon*. Proc Natl Acad Sci. 2016; 201610110. 10.1073/pnas.1610110114 27994146 PMC5224388

[10] Adhikari PL, Maiti K, Overton EB, Rosenheim BE, Marx BD. Distributions and accumulation rates of polycyclic aromatic hydrocarbons in the northern Gulf of Mexico sediments. Environ Pollut. 2016; 212:413–23. 10.1016/j.envpol.2016.01.064 26895564

[11] Pendergraft MA, Dincer Z, Sericano JL, Wade TL, Kolasinski J, Rosenheim BE. Linking ramped pyrolysis isotope data to oil content through PAH analysis. Environ Res Lett. 2013; 8(11):44038–10. 10.1088/1748-9326/8/4/044038

[12] Pendergraft MA, Rosenheim BE. Varying relative degradation rates of oil in different forms and environments revealed by ramped pyrolysis. Environ Sci Technol. 2014; 48(18):10966–74. 10.1021/es501354c 25105342

[13] Rosenheim BE, Day MB, Domack E, Schrum H, Benthien A, Hayes JM. Antarctic sediment chronology by programmed-temperature pyrolysis: Methodology and data treatment. Geochemistry, Geophys Geosystems. 2008; 9(4):1–16. 10.1029/2007GC001816

[14] Plante AF, Fernández JM, Haddix ML, Steinweg JM, Conant RT. Biological, chemical and thermal indices of soil organic matter stability in four grassland soils. Soil Biol Biochem. 2011; 43(5):1051–8. 10.1016/j.soilbio.2011.01.024

[15] Aeppli C, Carmichael C a., Nelson RK, Lemkau KL, Graham WM, Redmond MC, et al Oil weathering after the Deepwater Horizon disaster led to the formation of oxygenated residues. Environ Sci Technol. 2012; 46(16):8799–807. 10.1021/es3015138 22809266

[16] Ruddy BM, Huettel M, Kostka JE, Lobodin VV, Bythell BJ, McKenna A, et al Targeted petroleomics: Analytical investigation of Macondo well oil oxidation products from Pensacola Beach. Energy & Fuels 2014; 28(6): 4043–4050. 10.1021/ef500427n

[17] Redmond MC and Valentine DL. Natural gas and temperature structured a microbial community response to the Deepwater Horizon oil spill. Proc. Natl. Acad. Sci. 2012, 109, 20292–20297. 10.1073/pnas.1108756108 21969552 PMC3528494

[18] Dubinsky EA, Conrad ME, Chakraborty R, Bill M, Borglin SE, Hollibaugh JT, et al Succession of hydrocarbon-degrading bacteria in the aftermath of the Deepwater Horizon oil spill in the gulf of Mexico. Environ. Sci. Technol. 2013; 47, 10860–10867. 10.1021/es401676y 23937111

[19] Mason OU, Hazen TC, Borglin S, Chain PS, Dubinsky EA, Fortney JL, et al Metagenome, metatranscriptome and single-cell sequencing reveal microbial response to Deepwater Horizon oil spill. ISME J 2012; 6, 1715–1727, 10.1038/ismej.2012.59 22717885 PMC3498917

[20] White HK, Reddy CM, Eglinton TI. Isotopic constraints on the fate of petroleum residues sequestered in salt marsh sediments. Environ. Sci. Technol. 2005; 39(15), 2545–2551. 15884347 10.1021/es048675f

[21] White HK; Reddy CM; Eglinton TI. Radiocarbon-based assessment of fossil fuel derived contaminant associations in sediments. Environ. Sci. Technol. 2008, 42(15), 5428–5434. 18754456 10.1021/es800478x

[22] Reddy CM, Pearson A, Xu L, McNichol A, Benner BA, Wise SA, et al Radiocarbon as a tool to apportion the sources of polycyclic aromatic hydrocarbons and black carbon in environmental samples. Environ. Sci. Technol. 2002; 36, 1774–1782. 11998834 10.1021/es011343f

[23] Mason OU, Scott NM, Gonzalez A, Robbins-Pianka A, Bælum J, Kimbrel J, et al Metagenomics reveals sediment microbial community response to Deepwater Horizon oil spill. ISME J, 2014; 8(7), 1464–1475. 10.1038/ismej.2013.254 24451203 PMC4069396

[24] Pearson A, McNichol AP, Schneider RJ, von Reden KF, Zheng Y. Microscale AMS ^14^C measurement at NOSAMS. Radiocarbon. 1998; 40:61–75.

[25] von Reden KF, Donoghue JC, Elder KL, Gagnon AR, Gerlach DS, Griffin VS, et al Plans for expanded ^14^C analyses at the NOSAMS facility–A status and progress report. Nucl Instruments Methods Phys Res, 2004; 223–224, 50–54

[26] Longworth BE, von Reden KF, Long P, Roberts ML. A high output, large acceptance injector for the NOSAMS Tandetron AMS System, Nucl Instruments Methods Phys Res, 2015; 361, 211–2016

[27] Hemingway JD, Galy VV, Gagnon AR, Grant KE, Rosengard SZ, Soulet G, et al Assessing the blank carbon contribution, isotope mass balance, and kinetic isotope fractionation of the ramped pyrolysis/oxidation instrument at NOSAMS. Radiocarbon, 2017; 59(1), 179–193. 10.1017/RDC.2017.3

[28] Choi Y, Wang Y, Dynamics of carbon sequestration in a coastal wetland using radiocarbon measurements. *Global Biogeochem*. *Cycles*, 2004; 18 (4), 1–12. 10.1029/2004GB002261

[29] Fernández-Carrera A, Rogers KL, Weber SC, Chanton JP, Montoya JP. Deep Water Horizon oil and methane carbon entered the food web in the Gulf of Mexico. Limnol. Oceanogr. 2016, 61, S387–S400. 10.1002/lno.10440

[30] Atlas RM, and Hazen TC. Oil biodegradation and bioremediation: a tale of the two worst spills in U.S. history. Environ Sci Technol, 2011; 45(16), 6709–15. 10.1021/es2013227 21699212 PMC3155281

[31] Diercks AR, Highsmith RC, Asper VL, Joung DJ, Zhou Z, Gou L, et al Characterization of subsurface polycyclic aromatic hydrocarbons at the Deepwater Horizon site. Geophys Res Lett. 2010; 37(L20602) 10.1029/2010GL045046

[32] Zhou Z, Guo L, Shiller AM, Lohrenz SE, Asper VL, Osburn CL. Characterization of oil components from the Deepwater Horizon oil spill in the Gulf of Mexico using fluorescence EEM and PARAFAC techniques. Mar Chem. 2013; 148(0) 10–21

[33] Passow U, Ziervogel K, Asper V, Diercks A. Marine snow formation in the aftermath of the Deepwater Horizon oil spill in the Gulf of Mexico. Environ Res Lett, 2012; 7(3), 1–11. 10.1088/1748-9326/7/3/035301

[34] Passow U. Formation of rapidly-sinking, oil-associated marine snow. Deep Res Part II Top Stud Oceanogr, 2016; 129, 232–240. 10.1016/j.dsr2.2014.10.001

[35] Valentine DL, Kessler JD., d MC, Mendes SD, Heintz MB, Farwell C, et al Propane respiration jump-starts microbial response to a deep oil spill. *Science (New York*, *N*.*Y*.*)*, 2010; 330(6001), 208–11. 10.1126/science.1196830 20847236

[36] Reddy CM, Arey S, Seewald JS, Sylva SP, Lemkau KL, Nelson RK, et al Composition and fate of gas and oil released to the water column during the Deepwater Horizon oil spill. Proc Natl Acad Sci, 2012; 109(50): 20229–20234. 10.1073/pnas.1101242108 21768331 PMC3528605

[37] Diercks AR, Dike C, Asper VL, DiMarco SF, Chanton JP, Passow U. Resuspension scales in the northern Gulf of Mexico. Elem Sci Anth. 2018; 6(1):32 10.1525/elementa.285

[38] Chanton JP, Giering SL Bosman S, Rogers K, Sweet J, Asper V, et al Isotope Composition of Sinking Particles: Oil Effects, Recovery and Baselines in the Gulf of Mexico, 2010–2015. Elem Sci Anth. 2018; 6, 43 10.1525/elementa.298

[39] Wilkes H, Boreham C, Harms G, Zengler K, Rabus R. Anaerobic degradation and carbon isotopic fractionation of alkylbenzenes in crude oil by sulphate-reducing bacteria. *Org Geochem*, 2000; 31(1), 101–115. 10.1016/S0146-6380(99)00147-3

[40] Griebler C, Safinowski M, Vieth A, Richnow HH, and Meckenstock RU. Combined Application of Stable Carbon Isotope Analysis and Specific Metabolites Determination for Assessing In Situ Degradation of Aromatic Hydrocarbons in a Tar Oil-Contaminated Aquifer. Environ Sci Technol, 2004; 38(2), 617–631. 10.1021/es0344516 14750740

[41] Sun Y, Chen Z, Xu S, Cai P. Stable carbon and hydrogen isotopic fractionation of individual n-alkanes accompanying biodegradation: Evidence from a group of progressively biodegraded oils. Org Geochem, 2005; 36(2), 225–238. 10.1016/j.orggeochem.2004.09.002

[42] Wynn JG. Carbon isotope fractionation during decomposition of organic matter in soils and paleosols: Implications for paleoecological interpretations of paleosols. Palaeogeogr, Palaeoclimatol, Palaeoecol, 2007; 251(3–4), 437–448. 10.1016/j.palaeo.2007.04.009

[43] Krull ES, Bestland EA, Gates WP, Soil organic matter decomposition and turnover in a tropical Ultisol: Evidence from δ^13^C, δ^15^N and geochemistry. Radiocarbon. 2002; 44, 93–112

[44] Krull ES, Skjemstad JO, Burrows WH, Bray SG, Wynn JG, Bol R, et al Recent vegetation changes in central Queensland, Australia: evidence from δ^13^C and 14C analyses of soil organic matter. Geoderma 2005; 126(3–4), 241–259

[45] Wynn JD, Harden JW, Fries TL. Stable carbon isotope depth profiles and soil organic carbon dynamics in the lower Mississippi Basin. Geoderma 2006;131, 89–109.

[46] Chanton JP, Cherrier J, Wilson RM, Sarkodee-Adoo J, Bosman S, Mickle A. Radiocarbon evidence that carbon from the Deepwater Horizon spill entered the planktonic food web of the Gulf of Mexico. Environ Res Lett, 2012; 7 10.1088/1748-9326/7/4/045303

[47] Cherrier J, Sarkodee-Adoo J, Guilderson TP, Chanton JP. Fossil Carbon in Particulate Organic Matter in the Gulf of Mexico following the Deepwater Horizon Event. Environ Sci Technol Lett, 2014; 1(1), 108–112. 10.1021/ez400149c

[48] Daly KL, Passow U, Chanton J, Hollander D. Assessing the impacts of oil-associated marine snow formation and sedimentation during and after the Deepwater Horizon oil spill. Anthropocene, 2016; 13, 18–33. 10.1016/j.ancene.2016.01.006

[49] Montagna PA, Baguley JG, Cooksey C, Hartwell I, Hyde LJ, Hyland JL, et al Deep-Sea Benthic Footprint of the Deepwater Horizon Blowout. PLoS ONE, 2013; 8(8). 10.1371/journal.pone.0070540 PMC3737147 23950956

[50] Baguley JG, Montagna PA, Cooksey C, Hyland JL, Bang HW, Morrison C, et al Community response of deep-sea soft-sediment metazoan meiofauna to the Deepwater Horizon blowout and oil spill. Mar Ecol Prog Ser, 2015; 528, 127–140. 10.3354/meps11290

[51] Goñi MA, Ruttenberg KC, Eglinton TI. Source and contribution of terrigenous organic carbon to surface sediments in the Gulf of Mexico. Nature, 1997; 389(6648), 275–278. 10.1038/38477

[52] Goñi MA, Ruttenberg KC, Eglinton TI. A reassessment of the sources and importance of land-derived organic matter in surface sediments from the Gulf of Mexico. Geochim Cosmochim Acta, 1998; 62(18), 3055–3075. 10.1016/S0016-7037(98)00217-8

[53] Gordon ES, and Goñi MA. Controls on the distribution and accumulation of terrigenous organic matter in sediments from the Mississippi and Atchafalaya river margin. *Mar Chem*, 2004; 92(1–4), 331–352. 10.1016/j.marchem.2004.06.035

